# Medical students’ knowledge of ultrasonography: effects of a simulation-based ultrasound training program

**DOI:** 10.11604/pamj.2018.30.122.14820

**Published:** 2018-06-13

**Authors:** Oguz Eroglu, Figen Coskun

**Affiliations:** 1 Kirikkale University Faculty of Medicine, Department of Emergency Medicine, Kirikkale, Turkey

**Keywords:** Medical student, simulation-based ultrasound training, curriculum

## Abstract

**Introduction:**

The use of simulation devices in medical education is becoming more prevalent with each passing day. The present study aimed to teach medical students to perform ultrasonography via a simulation-based ultrasound training program.

**Methods:**

The study was prospectively conducted on final year medical students who had not received previous ultrasound training and who came to the Emergency Department of the Kirikkale University Faculty of Medicine between July 2015 and July 2016. Ultrasound training was provided by two emergency department specialists who are qualified in this field. The training time was determined to be 20h (4h for theoretical lessons, 16h for hands-on). The students were evaluated by a theory test and practical application exam both before and after training.

**Results:**

Obtained were compared using the paired sample t-test, and p < 0.05 was considered to be significant. Results: Ninety-six final year medical students were included. Their mean age was 24.1 ± 2.1 years. The mean test score obtained in the theoretical exam before training was 7.9 ± 2.2, while that after training was 17.1 ± 1.6 (p < 0.0001). The mean score obtained in the practical application exam before training was 1.1 ± 0.9 points and that after training was 10.9 ± 0.2 points (p < 0.0001).

**Conclusion:**

Medical students can learn to use an ultrasound device within a short period of time via simulation-based training programs. New studies must be conducted for the inclusion of ultrasound training programs in the medical education curriculum.

## Introduction

Training programs with simulation devices have many advantages for trainers and trainees, and their use in every field has been becoming more prevalent each day. The use of simulation-based training devices in the field of medicine has become the most important way to inculcate safe and repeatable learning methods for doctors who learn *"primum non nocere"* as their first foundation [1]. The agenda in medical education is determined by considering the immediate and long-term needs of students [2, 3]. In our country, the use of simulation-based devices is non-existent and there is no official curriculum regarding the use of these devices. The first curriculum study regarding ultrasound training in the field of emergency medicine was conducted by Mateer et al in 1994 [4]. Today, scientific societies such as the American College of Emergency Physicians, Society for Academic Emergency Medicine, and American Board of Emergency Medicine provide up-to-date application of the curriculum regarding ultrasound in United States [5, 6]. In our country, ultrasound training for emergency medicine assistants is given by hospitals they work in and by associations specializing in emergency medicine [7]. However, this training is only for assistant doctors and is not for medical students. Medical students come across the ultrasound device during medical education in different clinics, such as radiology, obstetrics and gynecology, or cardiology clinics, but they do not receive adequate theoretical and practical application training regarding the use of the ultrasound device. This study aimed to develop the knowledge and skills of final year medical students regarding to perform via a simulation-based ultrasound training program.

## Methods

Following local ethics board approval (No: 2015-14/06), the study was conducted on final year medical students who came to the emergency department of the Kirikkale university faculty of medicine for a two-month rotation between July 2015 and July 2016 and who had not received prior ultrasound training. Informed consent forms were taken from all students before the study.

**Figure 1:**
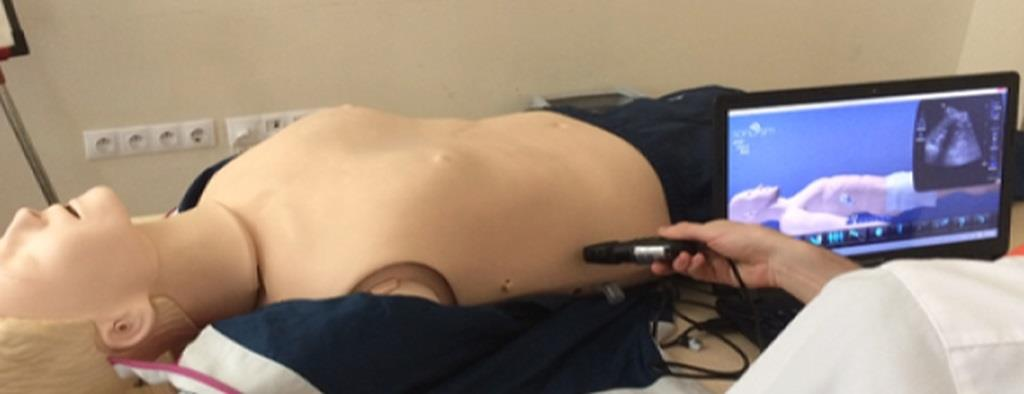
Practical application on a mannequin with a simulation-based ultrasound device

**Ultrasound training program**: Ultrasound training was provided over a 4-week period by two emergency medicine specialists who are qualified in ultrasound training. During the training period, 20 hour of ultrasound training was given with 1-hour-a-week theoretical class and 4-hour-a-week simulation-based practical application. Practical applications (hands-on) were carried out accompanied by the specialist trainer and using Sonosim ultrasound training solution device (Sonosim Inc. CA, USA). To make the hands-on training more realistic, a mannequin was used during the application (Figure 1). Practical applications were repeated until it was confirmed that each student held the ultrasound probe correctly, knew the ultrasound device, knew the anatomic tissue or organ, and differentiated pathological images. The ultrasound training program consisted of the following topics: basic ultrasound training (basic physics and knobology); focused assessment with sonography for trauma (FAST) exam: right upper quadrant, left upper quadrant, pelvic evaluation, and free fluid recognition; extended focused assessment with sonography for trauma (E-FAST) exam: thoracic evaluation and pneumothorax recognition in addition to the FAST exam; rapid ultrasound for shock and hypotension (RUSH) exam: evaluation of large blood vessel (inferior vena cava, internal jugular vein, aorta) and deep vein thrombosis in addition to the E-FAST exam; cardiac limited ultrasound examination (CLUE): cardiac cavity evaluation and cardiac tamponade recognition

**Theoretical (test) exam**: The same multiple choice questions were asked to the students before and after they received ultrasound training. There were 20 questions and their distribution was as follows: basic ultrasound training: 5, FAST exam: 8, E-FAST exam: 2, RUSH exam: 2, CLUE: 3 questions. Each correct answer was given 1 point, and each incorrect answer was given 0.

**Practical application exam**: The practical application exam was administered before and after training via the simulation-based ultrasound device. During the practical application exam, the students were asked to perform 12 different practical applications regarding knowing the ultrasound device, choosing the correct transducer, correctly holding the transducer, making the image clearer, evaluating the anatomic organ, cavities and quadrants, evaluating organ injuries and vessels, recognizing the heart and its cavities, and recognizing pathologies such as free fluid, cardiac tamponade, pneumothorax, and hemothorax. Each correct application was given 1 point, while each incorrect application was given 0 points.

**Statistical analysis**: All data were analyzed by SPSS 23.0 (Statistical Package for the Social Sciences Inc; Chicago, IL, USA) software. Variables were expressed as number (n) and percentage (%), and results are expressed as mean ± standard deviation (SD). The paired sample t-test was used in the comparison of results before and after training, and p < 0.05 was considered to be significant.

## Results

In total, 102 final year medical students participated; however, the study finally included 96 students after six students who did not attend the training programs regularly were excluded. The average age of the students was 24.1 ± 2.1 years, and 57.3% (n = 55) of the students were female. The mean test score of the students before training was 7.9 ± 2.2, while that after training was 17.14 ± 1.61 (p < .0001; Table 1). The mean practical application exam of the students before training was 1.1 ± 0.9, while that after training was 10.9 ± 0.2 (p < .0001; Table 2).

**Table 1:** Comparison of test results before and after training

Distribution of test questions	Number of questions	Test results		*p*
		Before training mean ± SD	After training mean ± SD	
Basic ultrasound physics	5	2.7 ± 0.9	4.7 ± 0.3	.0001
FAST exam	8	2.8 ± 1.3	7.2 ± 0.6	.0001
E-FAST exam	2	0.8 ± 0.7	1.7 ± 0.5	.0001
RUSH exam	2	0.3 ± 0.1	1.6 ± 0.2	.0001
CLUE	3	0.6 ± 0.2	1.9 ± 0.7	.0001
Total	20	7.9 ± 2.3	17.1 ± 1.6	.0001

FAST: focused assessment with sonography for trauma; E-FAST: extended focused assessment with sonography for trauma; RUSH: rapid ultrasound for shock and hypotension; CLUE: cardiac limited ultrasound examination; SD: standard deviation

**Table 2:** Comparison of practical application exam results before and after training

	Practical application exam				*p*
	Before training		After training		
	Correct ones		Correct ones		
Practical application required from the student	n	%	n	%	
Knowing the ultrasound device and its functions	3	3.1	95	98.9	<.0001
Choosing the transducer and adjusting the position	72	75	96	100	<.0001
Showing the liver, right kidney, hepatorenal cavity, and diaphragma	-	-	96	100	<.0001
Showing the spleen, left liver, splenorenal cavity, and diaphragma	-	-	94	97.9	<.0001
Showing the pelvic region, bladder, and/or Douglas’ pouch	1	1.1	95	98.9	<.0001
Showing the aorta, inferior vena cava, and internal jugular vein	-	-	86	89.6	<.0001
Showing the heart cavities and other cardiac structures	-	-	88	91.6	<.0001
Identifying intra-abdominal free fluid	1	1.1	95	98.9	<.0001
Identifying the pericardial tamponade	-	-	91	94.9	<.0001
Identifying pleural effusion and/or hemothorax	-	-	84	87.5	<.0001
Showing pleural gliding movement and identifying pneumothorax	-	-	93	96.9	<.0001
Identifying deep vein thrombosis	-	-	92	95.8	<.0001

## Discussion

The results of our study show that medical students can learn how to use an ultrasound device even in a short period of time with simulation-based ultrasound training programs. This is consistent with previously conducted studies. Favot et al. provided ultrasound training on trauma and vascular intervention for 4 weeks to fourth year medical students who came for the emergency services as part of their rotation and compared their results to those obtained from emergency medicine assistants working in the same place. In the multiple choice test given to both groups, the success level of medical students was higher than that of emergency medicine assistants [8]. In a similar study, medical students were given web-based training consisting of 45 min of theoretical lessons and 3h of practical training in ultrasound physics, device training, FAST exam, and central venous catheterization with ultrasound, and significant differences were found in the results of the test conducted before and after the training [9]. Fox et al separated medical students who came to the emergency services as part of their rotation into two groups according to their rotation periods as 2 weeks and 4 weeks. After providing training regarding ultrasound protocols that are commonly used in emergency services such as trauma and invasive interventions, they compared the two groups in terms of the learning level at the end of training and the retention of learned information 6 months after the training. According to the results of this study, the success level of the group that received 4 weeks of training was higher both at the end of the training and after 6 months than that of the group that received 2 weeks of training [6]. We determined the ultrasound training period as 4 weeks in our study. Although successful results have been obtained in training studies conducted for shorter periods, we believe that long-term and successful ultrasound training should not be for less than 4 weeks. We selected the contents of the ultrasound training program from the ultrasound protocols regarding the evaluation of clinical patients who require quick diagnosis and treatment such as trauma and shock at the emergency services. The content of our program is similar to that in other studies in the literature, and we believe that invasive interventions with ultrasound can also be included in the training program.

When models are being chosen for ultrasound training programs, volunteers, patients, or cadaver models, which have standard anatomic characteristics, are used [10, 11]. However, it is not always possible to find an appropriate model, particularly for bedside ultrasound practice. Moreover, body characteristics (for example, morbid obesity) or clinical conditions may not always allow ultrasound to be performed in a patient who has educational symptoms or typical ultrasonography images. Thus, simulation-based ultrasound devices are valuable resources for all doctors conducting the training program and the only applicable and sustainable method for meeting the needs in both pathologic and normal anatomic models [12-14]. Additionally, simulation-based ultrasound devices provide a safe environment for users. With frequently repeated practice performed in this safe environment, shyness, which occurs during patient evaluation, decreases, while the correct and quick evaluation rate increases. This also increases the self-confidence and motivation of trainees [2, 3]. McGraw et al found that assistants who underwent venous catheterization training for using a simulation device were both faster and more successful in intervention operations they conducted after the training program [15]. In another study, Turner et al compared two groups that used and did not use a simulation device during their ultrasound training in terms of manner, confidence, knowledge, and skill levels after training and found the results of the group that received ultrasound training with the simulation-based device to be better in every category [16]. In studies evaluating centers in USA that provide ultrasound training, although it was reported that each center has a different training program, some centers follow the traditional curriculum while others follow the formal curriculum, and there is no consensus on training times and manners, it was found that ultrasound training was performed with the support of simulation-based devices in all these centers [17-20]. In our study, we supplemented the theoretical training provided to the students with the simulation-based practical training. We believe that these devices are essential tools for training because they are easily accessible at any time, they provide pathologic or normal images with clarity, and they provide a repeatable and safe environment for users. In our study, due to budgetary limitations, not all ultrasound modules in the simulation device used for the purpose of training were purchased. Thus, during the training of cardiac evaluation, the "Cardiac Limited Ultrasound Examination" protocol was used instead of Echocardiography.

## Conclusion

The obtained results show that medical students can learn to perform ultrasonography even within a short period of time because of simulation-based training programs. However, new studies are needed to make ultrasound training for medical students a part of the routine medical training curriculum in our country.

### What is known about this topic

- Ultrasonography is an indispensable diagnostic tool for emergency service doctors;
- There is no formal curriculum in many countries about medical students' of ultrasound education;
- It is not always possible to have a sample patient that can be examined during ultrasonography training.

### What this study adds

- Medical education should keep pace with change;
- New curriculum for ultrasonography education of medical faculty students should be established;
- Via simulation-based ultrasonography devices ultrasonography training can be taught in a short time.

## Competing interests

The authors declare no competing interests.
